# Deployment of an End-to-End Remote, Digitalized Clinical Study Protocol in COVID-19: Process Evaluation

**DOI:** 10.2196/37832

**Published:** 2022-07-29

**Authors:** Nicole Zahradka, Juliana Pugmire, Jessie Lever Taylor, Adam Wolfberg, Matt Wilkes

**Affiliations:** 1 Current Health Inc Boston, MA United States; 2 Current Health Ltd Edinburgh United Kingdom

**Keywords:** evaluation study, telemedicine, remote consultation, digital divide, research design, virtual clinical trial, decentralized, COVID-19, primary recruitment, social media, virtual care, heart rate, wearable, health care cost, health technology

## Abstract

**Background:**

The SARS-CoV-2 (COVID-19) pandemic may accelerate the adoption of digital, decentralized clinical trials. Conceptual recommendations for digitalized and remote clinical studies and technology are available to enable digitalization. Fully remote studies may break down some of the participation barriers in traditional trials. However, they add logistical complexity and offer fewer opportunities to intervene following a technical failure or adverse event.

**Objective:**

Our group designed an end-to-end digitalized clinical study protocol, using the Food and Drug Administration (FDA)–cleared Current Health (CH) remote monitoring platform to collect symptoms and continuous physiological data of individuals recently infected with COVID-19 in the community. The purpose of this work is to provide a detailed example of an end-to-end digitalized protocol implementation based on conceptual recommendations by describing the study setup in detail, evaluating its performance, and identifying points of success and failure.

**Methods:**

Primary recruitment was via social media and word of mouth. Informed consent was obtained during a virtual appointment, and the CH-monitoring kit was shipped directly to the participants. The wearable continuously recorded pulse rate (PR), respiratory rate (RR), oxygen saturation (SpO_2_), skin temperature, and step count, while a tablet administered symptom surveys. Data were transmitted in real time to the CH cloud-based platform and displayed in the web-based dashboard, with alerts to the study team if the wearable was not charged or worn. The study duration was up to 30 days. The time to recruit, screen, consent, set up equipment, and collect data was quantified, and advertising engagement was tracked with a web analytics service.

**Results:**

Of 13 different study advertisements, 5 (38.5%) were live on social media at any one time. In total, 38 eligibility forms were completed, and 19 (50%) respondents met the eligibility criteria. Of these, 9 (47.4%) were contactable and 8 (88.9%) provided informed consent. Deployment times ranged from 22 to 110 hours, and participants set up the equipment and started transmitting vital signs within 7.6 (IQR 6.3-10) hours of delivery. The mean wearable adherence was 70% (SD 19%), and the mean daily survey adherence was 88% (SD 21%) for the 8 participants. Vital signs were in normal ranges during study participation, and symptoms decreased over time.

**Conclusions:**

Evaluation of clinical study implementation is important to capture what works and what might need to be modified. A well-calibrated approach to online advertising and enrollment can remove barriers to recruitment and lower costs but remains the most challenging part of research. Equipment was effectively and promptly shipped to participants and removed the risk of illness transmission associated with in-person encounters during a pandemic. Wearable technology incorporating continuous, clinical-grade monitoring offered an unprecedented level of detail and ecological validity. However, study planning, relationship building, and troubleshooting are more complex in the remote setting. The relevance of a study to potential participants remains key to its success.

## Introduction

Clinical researchers have leveraged emerging technologies to increase study efficiency and accuracy for over 20 years [1]. Digitalized and decentralized clinical study design allows investigators to recruit more heterogeneous populations, reduce the burdens of participation, and capture the experience of participants in real-world settings [2-4]. The SARS-CoV-2 (COVID-19) pandemic may accelerate the shift toward digitalized and decentralized clinical studies [5], forcing researchers to implement remote solutions that limit in-person interaction, while preserving clinical study integrity [1,5]. These mitigations are essential to prevent clinical study disruption, which have detrimental immediate and long-term effects on outcomes, treatment, and cost [6].

Technology already exists to enable digitalization of most aspects of a successful clinical study from recruitment through outcome collection [7]. Online platforms, such as social media, have been shown to be time-efficient and cost-effective methods of recruitment [2,8]. Teleconsent coupled with e-consenting resources can ensure the 3 elements of consent (information, comprehension, and voluntariness) are met, and have moderate-to-high levels of user satisfaction and ease of use across different populations [3]. Remote data collection tools range from online platforms and custom apps for self-reporting outcomes to wearables that continuously collect physiological measurements [4]. Such observations can be collected at high frequencies, increasing the granularity of data to improve the capture of clinically relevant outcomes in ecologically valid settings, compared to traditional clinical studies that typically involve less frequent observations collected during in-person study visits [9,10]. Ultimately, continuously worn wearable data sources may enable digital biomarkers and predictive models that translate detailed data into trial endpoints, clinically actionable insights, and effective diagnoses [11-13].

A fully remote clinical study protocol requires consideration of external factors that have typically been more easily eliminated, or controlled for, in traditional protocols. Strategies for participant education and “nudges” must be adapted for digital delivery when the underlying research question relies on the data and is not focused on capturing voluntary engagement with the data collection instruments. There is less participant visibility and fewer opportunities to intervene and correct during remote data collection compared to in-person study visits. Remote observational studies, therefore, are less reliable because data collected in this manner are more vulnerable to inconsistency and reliant on participant compliance. The effect of human-computer interaction on data collection, for better or for worse, must be considered during analysis. To an extent, these effects can be monitored (or at least, contextualized) by collecting measures of adherence alongside the primary study outcomes. Logistical considerations, such as equipment shipment duration, become factors when eligibility and data collection are time sensitive or following a technical failure or adverse event.

In this study, we designed an end-to-end digitalized clinical study protocol using the Current Health (CH) remote monitoring platform (Current Health Ltd.) to collect symptoms and continuous physiological data to build novel predictive algorithms of COVID-19 progression and severity in individuals who were recently infected in the community. Risk scores based on demographics and risk scores for hospitalized patients already exist [14]. However, by combining continuous remote patient monitoring with machine learning, the goal was to predict the risk of hospitalization, intensive care unit (ICU) treatment, or death for an individual infected with COVID-19 based on their vital signs while still in the community. The CH wearable and software platform were Food and Drug Administration (FDA) 510(k)–cleared for vital sign collection; therefore, confidence in the quality of vital sign observations captured by the CH wearable was higher than the unregulated general wellness wearables that are commercially available [9]. It was hoped that future CH platform integration of the risk algorithm developed from the vital sign data collected in this clinical study might improve resource allocation for patients after a COVID-19 diagnosis and enable more patient-centered management, increasing confidence for low- and high-risk patients and for those managing their care.

Given the need to recruit individuals positive for COVID-19 within 48 hours, key recruitment methods were social media and word of mouth. Recruitment through social media facilitated rapid reach, to an audience most likely to be eligible, in geographical locations associated with high numbers of COVID-19–positive cases and low vaccination rates [15-17].

Two recruitment methods, in person and pairing with test centers, were considered but not pursued. In-person recruitment was eliminated due to the increased risk of exposure and transmission of the infectious disease to the study team. Test center pairing was explored but was unsuccessful because test sites already had existing partnerships with academic institutions or were discouraged from advertising research studies that might deter COVID-19 testing.

Conceptual recommendations for digitalized and remote clinical studies have been outlined in the literature [7,18,19]. However, detailed examples, reviews, and learnings from actual implementation of these recommendations are limited. Our study implemented best-practice recommendations, although we were unable to progress beyond the pilot stage due to low recruitment. The goal of this paper is to describe the study setup in detail, evaluate its performance, and identify points of success and failure.

## Methods

### Trial Methodology

A scalable end-to-end digitalized protocol was designed for an observational clinical study in individuals who tested positive for COVID-19 in the community. Following the pilot phase, target recruitment was to be 2000 participants and enrollment was time sensitive following a positive COVID-19 test. The protocol eliminated in-person interaction and limited person-to-person interaction by utilizing commercially available technology**.** Each part of the research study, including recruitment, screening, consent, equipment setup, data collection, and follow-up, was automated, when possible.

### Ethical Considerations

The study was advertised on social media platforms, including Facebook, Instagram, and LinkedIn, from March 2021 through May 2021. The language in the recruitment material ranged from general and inclusive, such as “positive COVID-19 test,” to emphasizing the time-sensitive inclusion criteria of “tested positive for COVID-19 in the past 48 hours” as a call to action (Multimedia Appendix 1). Emails were also distributed internally to employees of CH, and study information was shared with family and friends through word of mouth. The study was conducted according to the guidelines of the Declaration of Helsinki, and the study protocol and recruitment materials were approved by the Advarra Institutional Review Board (Protocol Pro00047371, December 15, 2020; Advarra, Columbia, MD, USA).

Advertisements and emails directed interested individuals to Community by Current Health, a central resource for all CH research studies [20]. The COVID-19 study page included the time-sensitive inclusion criteria (positive COVID-19 test in the past 48 hours), a study overview, frequently asked questions, and a button to “Volunteer Today,” which led to a web-based eligibility form (Jotform), and eligible subjects could schedule an appointment with the study team online. Individuals were also asked whether they were agreeable to be contacted for future studies. Eligibility questions were accompanied by a rule set based on inclusion/exclusion criteria (Multimedia Appendix 2) to automate most of the screening process. After the appointment was scheduled, the individual received a copy of the informed consent document. Study team members contacted eligible individuals using the contact number provided. If not eligible, a popup window indicated they were ineligible.

The virtual appointment with a study team member was typically the first point of interaction between the eligible individual and study team member. This appointment, while remote, was a chance to build rapport with the potential participant while taking them through the informed consent process. Teleconsent was similar to an in-person experience, where the details of the study, benefits, risks, and potential conflicts of interest were explained, and time was provided to address any questions [8]. If the eligible individual was still interested in participating, the informed consent form (ICF) was sent to them through software that enabled signature verification and was Health Insurance Portability and Accountability Act (HIPAA) compliant (DocuSign, Inc.). The consent process took approximately 10-15 minutes, and the study team received an updated ICF with the digital signature of the participant. The study team member who obtained the informed consent was designated as the point of contact with the participant throughout the study duration to maintain consistency and engagement. A central study team contact number was used to monitor communication and provide responses within 24 hours. The communication platform was flexible and included text, email, or phone contacts based on participant preference. A system was developed between the study team and the shipping team to track package movement. A logistics partner (Seko Worldwide LLC, Itasca, Illinois, USA) was engaged to distribute and return equipment. The tracking information was also sent to the participants to increase their engagement and to engender a sense of responsibility. Return labeling and packaging were provided. Once consent was obtained, study equipment was delivered to the participants within 1-2 business days.

### Data Collection

During the pilot phase, the CH remote monitoring platform was used to collect vital signs and symptoms from 8 individuals who tested positive for COVID-19 (mean age 35.6 years (SD 10 years); 6 (75%) female; 7 (87.5%) White non-Hispanic; 1 (12.5%) Black or African American) for up to 30 days. Study endpoints included recovery (as defined by the Centers for Disease Control and Prevention Clinical Criteria), hospitalization, or death [21]. Therefore, study participation duration varied (mean 27.1 days, SD 5.4 days). The CH kit included a clinical-grade wearable that continuously recorded pulse rate (PR), respiratory rate (RR), oxygen saturation (SpO_2_), skin temperature, and step count and a tablet configured to the local time zone of the participant that administered surveys and task reminders. Participants were spread across 3 US time zones (Eastern, Central, and Mountain). Vital signs collected by the wearable and survey responses recorded in the tablet were transmitted to the CH cloud-based platform as raw waveforms and displayed in the web-based dashboard where compliance to study procedures could be monitored. Vital signs were sent when the CH wearable was in range of the CH transmitter and stored for up to 10 hours on the wearable if out of range. The transmitter was enabled for both home Wi-Fi and cellular communication, broadening participation to those without home internet.

An email was sent parallel to study equipment delivery to prompt the participants to set up the equipment as soon as possible. The email contained recommendations regarding wearing and charging the wearable and answering the daily survey. There was a prompt to complete a welcome survey using a unique weblink (Jotform). The welcome survey included questions about the participant and took less than 5 minutes to complete (Multimedia Appendix 3). Although participants were encouraged to complete the welcome survey at the beginning of the study, survey responses were accepted at any point during the study duration.

The CH kit was set up independently by the participants in their home using the tablet-guided instructions. The study team was available to provide remote assistance if there were difficulties setting up the equipment. The participant wore the CH wearable on the upper arm. The PR, SpO_2_, motion, and skin temperature were recorded at up to 30 samples per minute, and RR was recorded at up to 15 samples per minute when the wearable was on-arm. Two notifications were received on the tablet each day at 9:00 a.m. and 10:00 a.m. (local time zone). The first was a reminder to charge the wearable, and the second was a reminder to complete a brief series of questions about symptoms and decisions each day (Multimedia Appendix 4). The participants were not able to see vital sign data or survey responses in real time. Participant adherence was remotely monitored by applying a threshold alarm to vital sign data. The study team were alerted when a participant did not transmit vital sign data to the CH platform for 4 or more hours. The study team escalated the alert by contacting the participant to see whether they were experiencing technical issues or were away from home or to remind the participant to wear the CH device. The vital signs were not monitored in real time, which was made explicitly clear during the consent process. Participants were encouraged to act as they normally would if they felt unwell. At the end of the study duration, the participants returned the CH kit via mail, receiving up to US $100 (US $25/week) if they successfully adhered to the study protocol, in recognition of their time.

### Evaluation Methodology

Metrics were created to quantify each phase of the study that was delivered remotely: recruitment, screening, consent, equipment setup, and data collection (Multimedia Appendix 5). Facebook advertising engagement and CH website traffic were tracked with a web analytics service (Google LLC). Advertisement clicks and website views were counted. Metrics of data collected during the trial from the 8 participants, such as daily wearable adherence, vital signs per day (PR, RR, SpO_2_), and symptoms, were calculated, in addition to quantitative metrics of trial evaluation. All metrics were assessed for distribution through visual inspection and the Shapiro-Wilk test. Where metrics were normally distributed, they are presented as the mean (SD), and where they were nonparametric (Shapiro-Wilk significant) they are summarized as the median (IQR).

## Results

### Enrollment Funnel

Of 13 different study advertisements, 5 (38.5%) were live on Facebook at any one time (Multimedia Appendix 1). There were 8852 clicks on the Facebook advertisements for a total spend of US $6770.35. Community by Current Health and its COVID-19 study page were viewed 8932 and 618 times, respectively. There was a decrease in the mean unique advertisement clicks per day from 100.83 in March 2021 to 28.97 in May 2021. In total, 38 eligibility forms were completed, and 19 (50%) respondents met the eligibility criteria. Of these, 9 (47.4%) were contactable (the remainder were uncontactable or deemed themselves “too sick” to take part). Informed consent was obtained from these 9 individuals, and 8 (88.9%) signed the ICF within 9 (SD 8) minutes of the study team sending the ICF via DocuSign (Table 1).

Deployment times ranged from 22 to 110 hours, with a median time of 41 (IQR 28-68) hours. Participants set up the study equipment and started transmitting vital signs within 7.6 (IQR 6.3-10) hours of delivery, and 5 (62.5%) of 8 participants completed the welcome survey in a median of 7.4 (IQR 7.2-53) hours of receiving the welcome email. In addition, 2 (25%) participants completed the welcome survey after the 30-day study period (time to task completion was 32 and 51 days, respectively), while 1 (12.5%) participant never completed the welcome survey. Welcome survey responses indicated that 7 (87.5%) participants did not have asthma, cancer, chronic obstructive pulmonary disease (COPD), diabetes, heart conditions, high blood pressure, sickle cell disease, or kidney disease; undergo an organ transplant; or take beta blockers. In addition, 3 (37.5%) of the participants were smokers, and 7 (87.5%) participants lived with other people but only 1 (12.5%) participant had another member in the household currently COVID-19 positive.

Study participation varied from 17 to 30 days, with 6 (75%) participants completing 30 days and 2 (25%) participants released at 17 and 20 days, respectively, having met the definition for recovery. The mortality rate was 0%, and none of the participants were hospitalized. The mean wearable adherence was 70% (SD 19%), and the mean daily survey adherence was 88% (SD 21%). The median daily wearable adherence ranged from 52% (IQR 29.2%-82.0%) to 92.7% (IQR 82.6%-96.1%). The median PR per day ranged from 65.4 (IQR 59.3-75.5) to 96.5 (IQR 89.9-100.1) beats per minute. The median RR per day ranged from 15.5 (14.1-18.2) breaths/min to 19.3 (15.9-23.0) breaths per minute. The median SpO_2_ per day ranged from 95.8% (IQR 93.0%-97.2%) to 98.0% (IQR 96.7%-98.5%). Reported symptoms decreased over time (Multimedia Appendix 6). Participants triggered 87 technical alarms (12 [13.8%], for low battery and 75 [86.2%], for no data for >4 hours, although in many cases the data were buffered and transfer resumed once they had returned home). The median alarms per patient per day was 0.68 (IQR 0.57-1.0, range 0.25-1.6); these were predominantly in the morning. In addition, 4 (50%) participants met the criteria for wearable adherence (wearable worn for at least 20 hours a day and at least 6 days a week, up to 30 days). However, 4 (50%) missed 2 consecutive days of surveys and 1 (12.5%) failed to return the kit at the end. So, 2 (25%) of 8 participants met the strict criteria for full adherence to the study.

**Table 1:** Enrollment funnel.

Enrollment step	Participants, n (%)
Assessed for eligibility (N=38)	38 (100)
Eligible (N=38)	19 (50)
Informed consent call (N=19)	9 (47.4)
Enrolled (N=9)	8 (88.9)
Completed trial (N=8)	8 (100)

## Discussion

### Principal Findings

This work evaluated the performance of an end-to-end digitalized clinical study implemented using best-practice recommendations. In total, 8 participants were enrolled into the study; symptoms and continuous physiological data were collected using the CH platform for up to 30 days. Metrics associated with enrollment, deployment, and adherence to the study procedures were reported, and study data were summarized per day. During study participation, vital signs remained within their normal ranges and symptoms decreased over time (Multimedia Appendix 6). This study demonstrated both the advantages and the compromises inherent in decentralized, digitalized clinical trials, and we hope this experience will be valuable to other research groups considering a similar approach.

Throughout the trial, communication strategies were optimized to maintain engagement with participants without being invasive or time-consuming. A central number was created via Google Voice so that multiple study team members could be in communication with 1 participant from the same source. Email communication was timed around equipment delivery dates, and study milestones, to offer avenues for support at the times when most likely to be needed. Regular contact, by multiple channels, helped maintain engagement and reduced the likelihood of a participant being lost to follow-up. The study spanned several time zones, and rather than being a hindrance, this facilitated recruitment as study staff could offer longer “office hours” for participants. Investigators were nonetheless conscious to strike a balance between forming a relationship with a participant but allowing them sufficient space and anonymity for participant to re-engage if they forgot a study task.

The study logistics had to run parallel with participant communication. Engaging a logistics partner offered a level of flexibility in returning study equipment, and the participants were only paid their accrued study incentives once the equipment had been safely received. Collectively, these measures were reflected in most participants receiving equipment and beginning to transmit data within 48 hours of consent and all but 1 of the kits being returned at the close.

Decentralized, digitalized clinical trials also have unique challenges. Study planning, including multidisciplinary working and participant review, can either be facilitated or made more complex, depending on the circumstances of the work. All software must be HIPAA-compliant. Electronic documents must be organized into a study master file that maintains collaboration but also the integrity of study and participant confidentiality. This is typically accomplished by keeping participant information separate from study identifiers. In the CH platform, a random token unique to each participant was generated and stored along with personally identifiable information (PII) in a secure PII enclave. All clinical data processing was then performed on de-identified data, including only that token.

Wearable devices must be validated in the study setting, including being used by the population in question without contemporaneous instruction, and there should be appropriate internet or cell coverage for data transmission (which may exclude certain populations). Wearable continuous monitoring provides vastly more data than single study visits, so consideration should be given to data storage, triage, and quality. A decision must be made a priori between storing raw data, which maintains maximum fidelity and flexibility for future investigation (but is costly), and committing to a level of aggregation. One argument for choosing wearables that record and store raw waveforms is that wearable sensors vary in quality (only some have achieved FDA 510k clearance for clinical-grade monitoring) and participants are out of sight of investigators. Storing the raw waveforms allows for retrospective audit of vital sign quality, which can be reassuring. At the very least, devices should be selected that supply an indication of data quality as metadata.

Given this potential to collect vast amounts of data, definitions of adherence must be set around the goals of the study and be realistic for the participants throughout their participation. More data are not always more informative, but more of the “right data” will be. Our definition of full adherence was strict, with a high bar set for wearable adherence and survey completion. Although this bar may have been appropriate and achievable in the more severe phases of the illness, once participants felt well again, they started to leave home and re-engage with work, and it proved too high for some. Equally, if technical alerts for no data are to be set, they should also be set at a threshold and cadence mindful of the study goals and likely participant behavior. Although a proportion of our no-data alarms reflected genuine technical difficulty or nonadherence, most were simply triggered by symptom-free participants leaving the house for more than 4 hours at a time.

A remote trial clearly facilitates some aspects of research in an infectious disease population and reduces the likelihood of disease transmission to participants and investigators. However, for those quarantining within their own homes, troubleshooting device issues can be made more complex. It can be hard for them to retrieve deliveries of study components when the initial delivery has been missed. Patients who are acutely unwell may be less likely to see study advertisements. As discussed earlier, they may also be harder to connect with or may feel too sick or disinclined to participate. This may skew results toward less severe illness. Infections, by their nature, are time sensitive. Logistical delays or issues with equipment may leave some participants ineligible. Relationship building can be more difficult to foster when the participant and investigator never meet in person. It is harder to gauge understanding during the informed consent process, and some study data, particularly sensitive information or demographic details, may be harder to acquire. A balance must be struck between creating appropriate minor hurdles to ensure the participant is serious about completing the study, and ensuring overall ease of participation and appropriate incentives to recognize the minor inconvenience associated with data collection.

### Comparison With Prior Work

Social media platforms hold much promise as avenues for recruitment. Their advertising models are designed and priced to target particular demographics in specific locations, and their advertisements are readily amenable to A-B testing. The platforms’ broad reach and pricing models such as cost-per-click offer fine control over costs and potential savings to investigators. Ali et al [2] reported enrollment of 6602 participants with 9609 advertisement clicks and a total spending of US $906 over the recruitment period. Although there were 8852 advertisement clicks (US $6770.35) in our study, enrollment was much lower (9 participants). However, without a bricks-and-mortar institution, online recruitment can still struggle to achieve a signal amidst the noise. In the context of COVID-19 studies and the pandemic, ring fencing of some language on the platforms made this signaling more challenging. For example, Facebook prohibits advertisements containing content that asserts or implies personal attributes, including physical health.

Indeed, in our study, the initial phase of recruitment remained the most difficult. Advertisements and their amendments were often delayed, while their content was manually reviewed for language. Although 19 participants were eligible, only 9 agreed to a phone call, with many citing a worsening of their condition as a reason not to participate. In studies of rapidly evolving diseases, such as COVID-19, it would be prudent to tie the process of information and consent directly to testing in order to enroll potential participants once proven positive but before their symptoms become overwhelming.

However, once the challenges of initial recruitment were overcome and participants were in the funnel, all patients except 1 who received a phone call consented to the study. This success at converting interest to consent and participation may have been because online recruitment removed perceived barriers to participation or because word of mouth was also used as a recruitment tool. A systematic review and meta-analysis of online patient recruitment in clinical trials found that traditional offline recruitment strategies (eg, word of mouth) result in higher conversion rates than online recruitment strategies [8]. Proximity to a study site was not required. There was no lead time to appointments, and scheduling and coordination of information sharing and consent were simplified. The economic burden on participants, in terms of travel, time, and opportunity cost, was reduced. In the context of COVID-19, there was no concern about viral transmission to participants or investigators from visiting an institution. Removing these barriers, real and perceived, may allow remote clinical trials to reach more marginalized communities, often the hardest for researchers to enroll and, in the case of COVID-19, disproportionately affected by the pandemic.

### Limitations

Ultimately, the perceived relevance of a study to its participants, and the landscape in which it is deployed, will hold the key to its success. This pilot study was opened in February 2021 just as infection rates were falling and vaccination rates were rising. Despite incorporating recommendations for best practices in decentralized trials, and our success at enrolling participants once in the funnel and removing barriers to participation, we still missed our recruitment targets and evaluation of the implementation of a scalable end-to-end digitalized protocol was limited to a small sample size. Decentralized and virtual studies hold enormous promise, and may indeed revolutionize the way studies are conducted, but they will still likely remain 1 of many tools in the clinical trials toolkit.

### Conclusion

Our pilot study demonstrated the advantages, challenges, and compromises inherent in digitalized, decentralized remote clinical trials. With a well-calibrated approach to online advertising and enrollment, barriers to recruitment can be removed and the cost reduced. Equipment can be effectively and promptly shipped to participants, without risk of illness transmission during a pandemic. Wearable technology incorporating continuous, clinical-grade monitoring can offer an unprecedented level of detail and ecological validity. However, study planning, relationship building, and troubleshooting are more challenging, and definitions of adherence must be crafted around anticipated participant behavior. The relevance of a study to potential participants, be it in person or remote, remains key to its success, particularly during a pandemic.

## Appendix

Multimedia Appendix 1 Social media.

Multimedia Appendix 2 Trial eligibility.

Multimedia Appendix 3 Welcome survey.

Multimedia Appendix 4 Daily survey.

Multimedia Appendix 5 Quantitative metrics of trial evaluation.

Multimedia Appendix 6 Metrics of data per day during the 30-day study duration. (A) Median (IQR) adherence per day. The red line indicates the number of participants enrolled on each day. (B) Symptom count per day collected from the daily survey administered on the CH tablet. The count is of the number of participants who reported the symptom. Colors represent different symptoms. The numeral above each bar represents the number of participants who completed the daily survey. (C-E) Median (IQR) vital signs per day collected by the CH wearable.

## Abbreviations

CH: Current Health
FDA: Food and Drug Administration
HIPAA: Health Insurance Portability and Accountability Act
ICF: informed consent form
PII: personally identifiable information
PR: pulse rate
RR: respiratory rate
SpO_2_: Oxygen saturation
