# Supplementary material for: Deployment of an End-to-End Remote, Digitalized Clinical Study Protocol in COVID-19: Process Evaluation
Source: JMIR Form Res. 2022 Jul 29;6(7):e37832. doi: 10.2196/37832 (PMC9345299; doi:10.2196/37832)
Supplement: Multimedia Appendix 1 [file formative_v6i7e37832_app1.pdf]

Appendix. Four examples of the 13 social media study advertisements. Other study ads were variations of the same language.

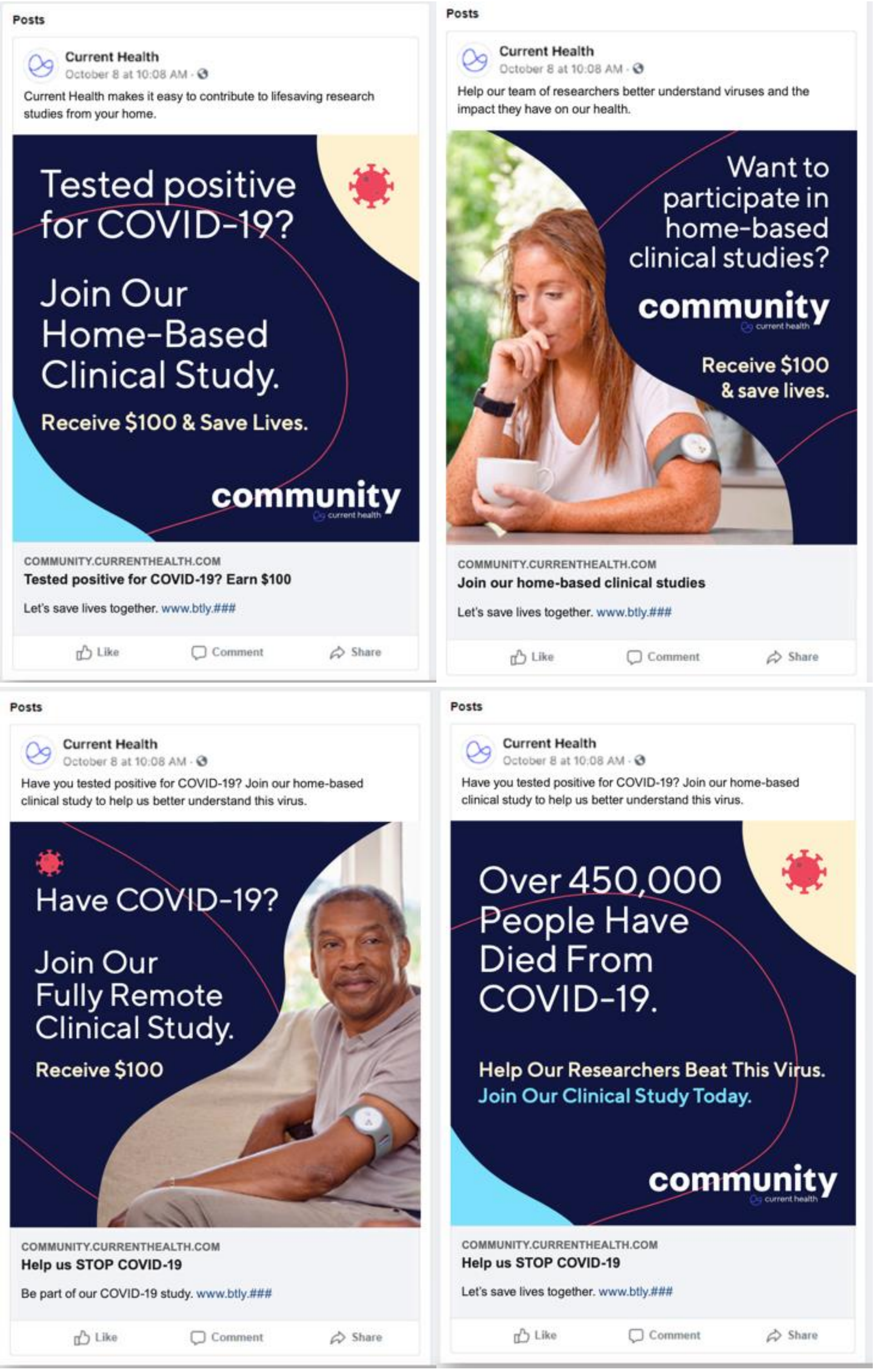

This is a Multimedia Appendix to a full manuscript published in the J Med Internet Res. For full copyright and citation information see http://dx.doi.org/10.2196/jmir.37832
