# Supplementary material for: Deployment of an End-to-End Remote, Digitalized Clinical Study Protocol in COVID-19: Process Evaluation
Source: JMIR Form Res. 2022 Jul 29;6(7):e37832. doi: 10.2196/37832 (PMC9345299; doi:10.2196/37832)
Supplement: Multimedia Appendix 2 [file formative_v6i7e37832_app2.pdf]

Trial Inclusion Criteria

Inclusion	Exclusion
Living in the United States (contiguous states)	Cannot confirm a positive PCR or antigen test for COVID-19
21 years or older	Unable to read English
Able to provide documentation of a positive PCR or antigen test for COVID-19 within the past 48 hours	Unwilling or unable to provide baseline data required for study entry
Self-reports comfortable and willing to wear the wearable device and interact with a tablet-based interface daily	Heavy tattooing on both upper arms (affected wearable sensor performance)
Able to provide a next of kin/designated person who can be contacted in the event of hospitalization for follow up	Known persistent atrial fibrillation (affected wearable sensor performance)
	Has taken/is taking part in a COVID-19 vaccine or treatment trial
