# Supplementary material for: Deployment of an End-to-End Remote, Digitalized Clinical Study Protocol in COVID-19: Process Evaluation
Source: JMIR Form Res. 2022 Jul 29;6(7):e37832. doi: 10.2196/37832 (PMC9345299; doi:10.2196/37832)
Supplement: Multimedia Appendix 3 [file formative_v6i7e37832_app3.pdf]

Appendix. Welcome Survey questions

Welcome!
Today is your first day in Community by Current Health's COVID-19 Study. We need to collect some information about you. We will only ask you these questions one time. This survey should take less than 5 minutes to complete.

Thank you again for taking part in this study. With your help our researchers will gain a better understanding of COVID-19 and be able to predict recovery and progression.

1) What is your date of birth?

2) What is your sex? Male/Female/other or prefer not to specify
    1) [IF FEMALE]
    2) To your own knowledge are you currently pregnant?
    3) Do you have menstrual periods?* Why do we ask this? Throughout the month, your body undergoes hormonal changes that affect factors such as your resting heart rate and body temperature that can potentially alter vital sign readings.
    4) Are you:
        - Pregnant or breastfeeding
        - Taking hormonal birth control
        - Undergoing menopause or post-menopausal
        - Highly active (working out, training, fitness, etc.)
    5) When was your last period?

3) What is your occupational category?
    Healthcare (doctor, nurse, dentist, etc.)
    Service industry (food service, grocery, mechanic, plumber, etc.)
    Retail
    Office – able to work from home
    Office – unable to work from home
    Currently unemployed
    Student – remote
    Student – attending class in person

4) What is your household make-up?
    Living alone
    Living with people

5) Do other members of your household currently have COVID-19? Y/N

6) Are any other members of your household enrolled in Community by Current Health's COVID-10 Study? Y/N

7) What is your self-identified race/ethnicity?
    White Hispanic
    White Non-Hispanic
    Black or African American
    Asian
    American Indian or Alaska Native
    Native Hawaiian and Pacific Islander

8) What is your height?

9) What is your weight (in pounds)?

Do you have any of the following conditions?

	Yes	No
Asthma		
Cancer		
COPD		
Diabetes		
Heart Condition		
High Blood Pressure		
Sickle Cell Disease		
Kidney Disease or reduced kidney function		

10) Have you ever had an organ transplant? Y/N

11) Do you take **beta blockers**? these are medicines designed to reduce heart rate, which can affect vital sign readings. For example: Tenormin, Cardiacor, Emcor, Betaloc, Inderal, or Angilol. Y/N

12) Are you a **smoker**? Y/N

13) Please enter any feedback about this survey:

This is a Multimedia Appendix to a full manuscript published in the J Med Internet Res. For full copyright and citation information see http://dx.doi.org/10.2196/jmir.37832
