# Supplementary material for: Deployment of an End-to-End Remote, Digitalized Clinical Study Protocol in COVID-19: Process Evaluation
Source: JMIR Form Res. 2022 Jul 29;6(7):e37832. doi: 10.2196/37832 (PMC9345299; doi:10.2196/37832)
Supplement: Multimedia Appendix 4 [file formative_v6i7e37832_app4.pdf]

Appendix. Daily Survey questions

Each day Current Health will ask you a **brief series of questions** about symptoms and decisions that may be related to COVID-19. Your daily response and information collected from the wearable device will create the most accurate picture of your health. This survey should take **less than 5 minutes** to complete.

1) In the **past 24 hours,** did you have (or continue to have) any of the following symptoms:

   - loss of smell or taste
   - dry cough
   - sore throat
   - fever or feeling feverish
   - chills
   - muscle aches
   - diarrhea
   - nausea

- [FOR EACH YES] Is your <symptom> better or worse than yesterday?
  categorical scale: Much worse, Worse, The same, Better, Much better

2) Due to how you are feeling **today,** do you plan to speak to a health care provider?

3) Due to how you are feeling **today,** do you plan to go to the hospital?

4) Do you feel as though you have completely recovered from COVID-19 as of **today**?

   Are there any other symptoms or differences in health you would like to report?

   [IF YES] Please list other symptoms or differences in health you would like to report.

Thank you for completing your daily survey! Your responses have been recorded.

This is a Multimedia Appendix to a full manuscript published in the J Med Internet Res. For full copyright and citation information see http://dx.doi.org/10.2196/jmir.37832
