# Supplementary material for: Deployment of an End-to-End Remote, Digitalized Clinical Study Protocol in COVID-19: Process Evaluation
Source: JMIR Form Res. 2022 Jul 29;6(7):e37832. doi: 10.2196/37832 (PMC9345299; doi:10.2196/37832)
Supplement: Multimedia Appendix 5 [file formative_v6i7e37832_app5.pdf]

Quantitative metrics of trial evaluation

Metric	Definition	Results
Eligibility	Number of eligible individuals who completed an eligibility form, compared to the total number of completed eligibility forms.	19 out of 38 (50%)
Consent rate	Number of signed consent forms received compared to the total number of consent phone calls completed.	8 out of 9 (89%)
Deployment time	Difference in timestamps between receiving the signed consent form and delivering the study equipment.	41 (28-68) hours<br>Range 22-110 hours
Setup time	Difference in timestamps between delivery of the study equipment and first vital sign recorded.	7.6 (6.3-10) hours
Time to task completion	Difference in timestamps between the welcome email delivery and welcome survey completion.	7.4 (7.2-53) hours
Wearable adherence	Number of hours worn between the start of observations and their discharge from the study as a proportion of maximum wear, as percentage.	70 ± 19 %
Daily survey adherence	Number of daily surveys completed compared to the number of daily surveys assigned, as percentage.	88 ± 21 %
Full adherence	Wearable worn for at least 20 hours a day and at least six days a week, up to 30 days; daily survey responses at least 6 days a	25 % of participants

	week up to 30 days; Current Health kit returned following participation.
