# Supplementary figures and images for: Deployment of an End-to-End Remote, Digitalized Clinical Study Protocol in COVID-19: Process Evaluation
Source: JMIR Form Res. 2022 Jul 29;6(7):e37832. doi: 10.2196/37832 (PMC9345299; doi:10.2196/37832)

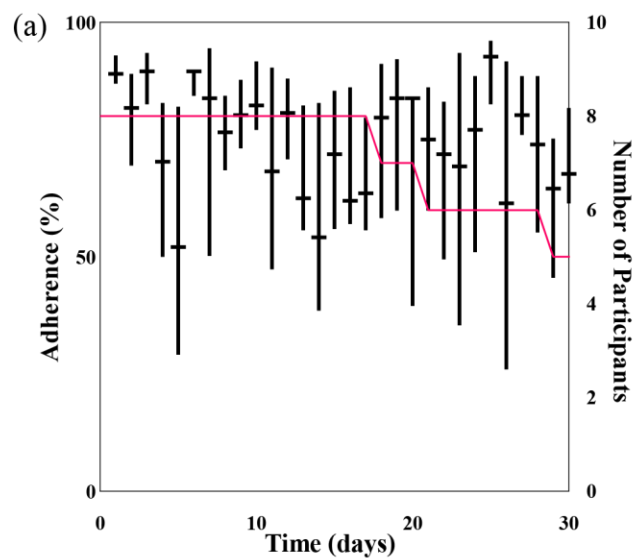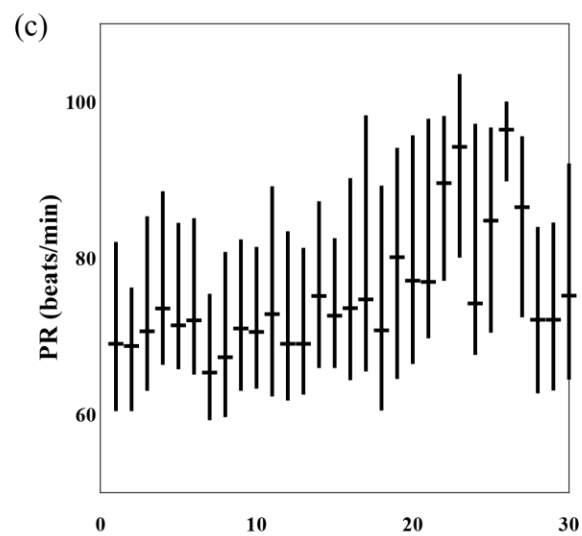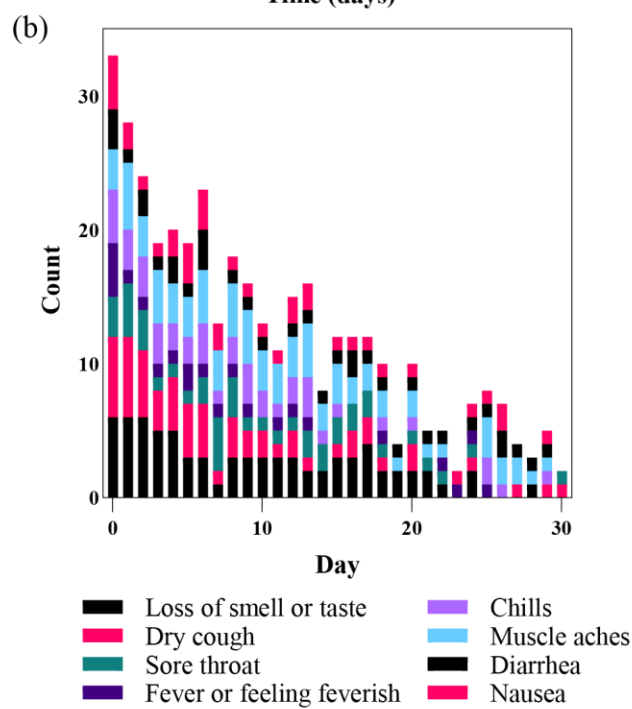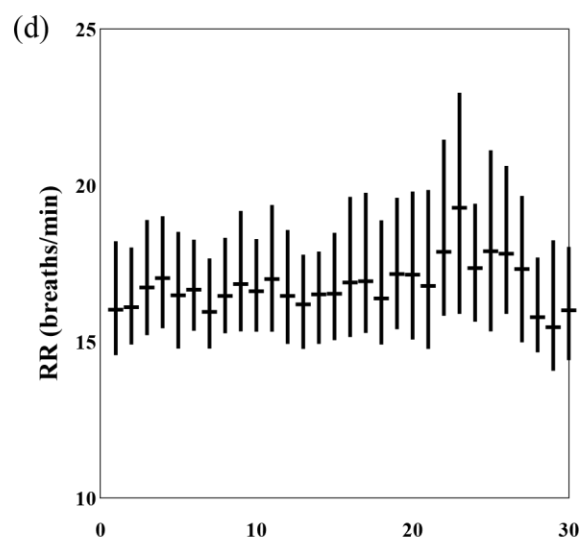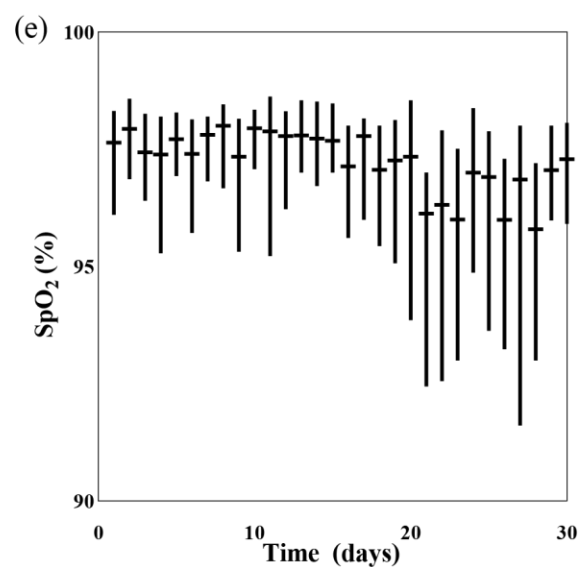

Supplement: Multimedia Appendix 6 [file formative_v6i7e37832_app6.pdf]
